# Pet Nutrition and the Unmeasured Owner Outcome: A Bidirectional Framework for Relational Value in Human–Animal Relationships

**DOI:** 10.3390/ani16182961

**Published:** 2026-09-20

**Authors:** Huakai Wang, Weiwei Wang, Qianqian Chen, Mengyao Ma, Yuqiang Zhang, Shiqiang Zhu, Ran Wang, Yinhua Zhu, Wei Xiong

**Affiliations:** 1 Food Laboratory of Zhongyuan, Luohe 462300, China; 2 Key Laboratory of Precision Nutrition and Food Quality, Department of Nutrition and Health, China Agricultural University, Beijing 100083, China

**Keywords:** pet health, nutritional intervention, relational value, human–animal bond, bidirectional dynamics

## Abstract

The emotional benefits of pet ownership depend critically on the pet’s physical, behavioural, and cognitive health—all of which can be improved by targeted nutritional interventions that may reduce pain, anxiety, problem behaviours, and cognitive decline. However, almost no studies have tested whether nutrition-induced behavioural improvements translate into measurable gains in owner emotional well-being or relationship satisfaction. It is important to note that the evidence base for cats remains considerably more limited than that for dogs, and many conclusions drawn from canine studies should be extrapolated to cats with caution. Furthermore, the human–animal relationship is bidirectional: owner psychology and feeding practices feed back to affect pet health. This review synthesises evidence across veterinary medicine, nutritional science, and human- animal interaction research to support a central thesis: that the relational value of pet ownership may be a function of pet health, may be modifiable by targeted nutrition, and is hypothesized to be embedded in bidirectional feedback loops that require dyadic interventions. The review explicitly distinguishes between evidence-supported findings and the proposed conceptual framework. The field now requires dyadic randomised controlled trials with co-primary outcomes for both pet and owner, plus precision stratification, pragmatic effectiveness studies, mechanistic biomarkers, and one health approaches. Closing this translational gap requires a coordinated research effort focused on designing dyadic interventions that measurably improve the welfare of both pets and their owners.

## Simple Summary

Owning a pet brings love and companionship, but these emotional benefits truly flourish only when the pet is physically and mentally healthy. If a pet suffers from pain, anxiety, behavioural issues, or cognitive decline, it can become a source of stress for the owner rather than comfort. This review explores how specific nutritional interventions—such as omega-3 fatty acids, probiotics, and functional ingredients—can improve a pet’s health and behaviour. A healthier pet with fewer behavioural problems allows for more joyful and positive daily interactions, which can directly ease the owner’s emotional burden. However, this relationship is a two-way street: an owner’s stress and feeding habits also affect the pet’s well-being, potentially creating a “vicious cycle.” We emphasize that future research must adopt a “dyadic” approach—measuring changes in both the pet’s behaviour and the owner’s mental health simultaneously. By breaking negative cycles and creating virtuous ones, nutritional strategies can ultimately enhance the lives of both pets and the millions of people who consider them family members.

## 1. Introduction

While hundreds of millions of households across the globe share their lives with companion animals, the well-documented emotional benefits of pet ownership—including reduced stress, alleviated loneliness, and improved overall well-being—are neither innate nor universally experienced by all owners [1,2,3]. A growing body of evidence indicates that the relational value owners derive from their pets depends critically on the pet’s physical, behavioural, and cognitive health [4,5]. A pet in chronic pain, suffering from a debilitating illness, exhibiting problem behaviours, or experiencing age-related cognitive decline may not only fail to provide consistent positive interaction but can itself become a source of owner distress, caregiver burden, and grief [6]. Conversely, when a pet’s health is actively supported, it can serve as a reliable and rewarding emotional partner. This recognition has recently drawn attention to nutrition as a modifiable determinant of pet behaviour and welfare. Moreover, the human–animal relationship is fundamentally bidirectional: owner psychological state, attachment style, and feeding practices feed back to affect pet health, creating self-reinforcing cycles.

This review proposes a paradigm shift in how we conceptualize the human–animal bond. We argue that the primary value of interventions such as nutrition must be measured by their direct impact on the animal’s own welfare, not merely as tools to satisfy owner needs. We therefore define ‘relational value’ as the quality of a co-produced bond where the animal’s intrinsic welfare (e.g., being free from pain, anxiety, and cognitive decline) and the owner’s psychological health (e.g., reduced burden, increased joy) are mutually constitutive and equally weighted. This framework explicitly rejects a transactional or extractive view of the animal. We propose a forward-looking research agenda that prioritises dyadic randomised controlled trials, precision stratification, pragmatic effectiveness studies, mechanistic biomarker validation, and integrated one health interventions. Throughout this review, we explicitly distinguish between two types of content: (1) evidence-supported findings, which are based on peer-reviewed empirical studies, and (2) the proposed conceptual model, which represents our interpretation of how these findings may be interconnected. Section 3 and Section 4 primarily synthesize evidence regarding pet health and nutritional interventions; Section 5 introduces the conceptual bidirectional framework linking owner and pet; and the Conclusion presents the translational implications and research agenda derived from this framework.

## 2. Review Methodology

To meet the journal’s requirements for transparency and reproducibility, this review details the search process, including the search tools used, exact search strings, and the number of hits returned for each query. A comprehensive literature search was conducted between January 2026 and June 2026 using PubMed and Google Scholar. PubMed served as the primary quantitative database, and Google Scholar was used as a supplementary tool to identify cross-disciplinary or grey literature not indexed in PubMed.

The following search strings were entered into PubMed (search date: June 2026): Search String 1: (“pet nutrition” OR “dog nutrition” OR “cat nutrition”) AND (“behavioural problems” OR “cognitive dysfunction” OR “anxiety” OR “aggression”)—Returned *n* = 16 results. Search String 2: (“dietary intervention” OR “omega-3” OR “probiotics” OR “tryptophan” OR “medium-chain triglycerides” OR “alpha-casozepine”) AND (“pet health” OR “pet behaviour” OR “osteoarthritis” OR “cognitive function”)—Returned *n* = 1567 results. Search String 3: (“owner well-being” OR “caregiver burden” OR “human–animal bond” OR “owner mental health”) AND (“pet health” OR “owner stress” OR “attachment” OR “grief”)—Returned *n* = 655 results. Search String 4: (“pet” OR “dog” OR “cat”) AND (“quality of life” OR “relational Value” OR “psychological well-being”) AND (“owner” OR “caregiver”)—Returned *n* = 510 results. Search String 5: (“behavioural assessment” OR “C-BARQ” OR “attachment scale” OR “caregiver burden scale”) AND (“dog” OR “cat” OR “pet”)—Returned *n* = 112 results. Search String 6: (“bidirectional” OR “dyadic” OR “mutual influence” OR “synchronized”) AND (“human–animal” OR “pet-owner” OR “owner-dog” OR “owner-cat”)—Returned *n* = 68 results. The individual PubMed searches returned a combined total of 2928 records. After combining these search strings using the OR operator and removing 41 duplicate records within PubMed, a total of 2887 unique records were retained for initial screening. Google Scholar yielded an additional 96 records not indexed in PubMed. After merging the two datasets and removing cross-database duplicates by manual screening of titles, authors, and DOIs, a combined total of 2983 unique records were obtained. Following relevance-based title and abstract screening against the pre-defined inclusion criteria, 126 peer-reviewed articles were ultimately included in the final synthesis.

Inclusion criteria comprised peer-reviewed original research articles and review articles that addressed: (i) the effects of nutritional interventions on pet physiology or behaviour; (ii) the impact of pet health or behaviour on owner psychological outcomes (e.g., stress, depression, caregiver burden, or grief); or (iii) the bidirectional dynamics of human–animal relationships. Exclusion criteria included non-peer-reviewed literature, studies not involving dogs or cats, commentary articles lacking empirical data, and book chapters.

Given the narrative scope of this review, a full systematic screening protocol (e.g., PRISMA) was not applied. Instead, the authors manually screened the titles of the retrieved records for their direct relevance to the pre-defined inclusion criteria and the thematic framework of this review. Where the relevance of a record could not be determined from the title alone, the abstract was consulted to make a final judgment. All included publications were analyzed and grouped thematically to facilitate discussion of: (i) the influence of pet physical health (e.g., pain, obesity, sensory decline) on interactive capacity; (ii) the impact of pet behavioural problems (e.g., aggression, separation anxiety) on owner burden; (iii) the effects of specific nutritional interventions (e.g., omega-3 fatty acids, probiotics, tryptophan, medium-chain triglycerides, alpha-casozepine) on pet behaviour; (iv) the feedback effects of owner psychological factors (e.g., attachment style, stress) on pet welfare; and (v) the mechanisms underlying bidirectional cycles (vicious vs. virtuous). As this study was based exclusively on previously published material, no ethical approval was required.

## 3. Pet Health Status as the Foundation of Its Capacity to Deliver Relational Value

This section evaluates the direct welfare implications of common health conditions for the animal, and their subsequent impact on the shared relationship. The central proposition here is that an animal in pain or cognitive decline is first and foremost harmed as a sentient being. This primary harm constitutes an intrinsic welfare violation. In a co-produced relationship, this direct harm may translate into a profound relational crisis for the owner, manifesting as caregiver burden and grief. Thus, addressing the animal’s health is not about maintaining a ‘tool’ for the owner, but about alleviating the animal’s suffering, which simultaneously upholds the integrity of the shared bond.

### 3.1. Physical Health, Pain, and the Loss of Interactive Capacity

Chronic pain, most commonly from osteoarthritis (OA), affects approximately 20–60% of dogs (depending on diagnostic criteria and population) and a similarly high proportion of cats, yet it remains substantially underdiagnosed and undertreated [7,8,9]. Pain consistently alters behaviour: affected animals may become irritable, resist handling, show aggression toward familiar people, and lose interest in play. An owner reaching out to stroke a painful dog may be met with a growl or a snap—responses that are easily misinterpreted as “behavioural issues” rather than expressions of suffering. Epidemiological data indicate that OA prevalence increases with age, and that owner recognition of pain-related behavioural changes is poor, which likely contributes to chronic under-treatment [10,11].

Obesity, which affects 20–59% of dogs and 11.5–63% of cats, depending on geographic region and diagnostic criteria, compounds this problem [12,13]. Obese pets have reduced exercise tolerance [14], a higher risk of OA [10], and often develop insulin resistance [13], all of which limit shared activities such as walking, retrieving, or chase games. Because shared physical activity is a primary domain through which many owners derive emotional satisfaction and health benefits from their pets [15], obesity-related inactivity reduces opportunities for such interactions, thereby lowering relational reward. Moreover, obesity is associated with altered affective states, including lethargy and anhedonia [16], further reducing the pet’s availability for positive engagement.

Sensory decline—progressive vision or hearing loss—is common in ageing pets and fundamentally alters communication. A dog that cannot see approaching hand gestures or hear a recall command may startle, become defensively aggressive, or appear unresponsive. Owners frequently misinterpret these changes as willful disobedience or reduced affection, leading to frustration, attempts at punitive training, and progressive erosion of the human–animal bond [17]. Physical health problems are therefore not merely medical issues; they may also be relational ones [18].

### 3.2. Problem Behaviours, Aggression, and Owner Distress

Problem behaviours in dogs often represent **significant underlying distress or suffering for the animal** (e.g., chronic anxiety, fear, or frustration) [18,19]. When these behaviours persist, they can impose a significant burden on the owner, contributing to frustration and caregiver burden, and increasing the risk of pet relinquishment [18,19]. A systematic review concluded that these behavioural issues reduce owners’ social interactions, heighten negative, high-arousal emotions (e.g., stress and frustration), increase caregiver burden, and exacerbate depression and anxiety [20]. The relationship is bidirectional: baseline owner mental health predicts subsequent problem behaviours, and baseline problem behaviours predict deterioration in owner mental health [21]. A four-week prospective study further identified between-person and within-person correlations between dog behavioural problems and six owner outcomes, including depression, anxiety, loneliness, and suicidal ideation, although cross-lagged effects did not reach significance in this short-term study. Therefore, bidirectionality cannot be considered an established fact; the broader bidirectional framework presented in Section 5 is a hypothesis-generating model that requires confirmation [20].

Owner attachment style moderates these dynamics. In a large Finnish sample, owner neuroticism and poor mental well-being were linked to anxious attachment to the pet, whereas pet problem behaviours were associated with owner avoidant attachment [22]. Consequently, an aggressive or fearful pet may lead to emotional distance from the owner, which in turn deprives the pet of positive input that could improve its behaviour, creating a self-perpetuating cycle. This interpretation is consistent with evidence that owner avoidant attachment is associated with separation-related disorder in dogs [23]. Problem behaviours are not merely training issues; they often reflect underlying physiological or emotional distress and exert clinically meaningful effects on the owner’s mental health [20].

### 3.3. Chronic and Terminal Illness: Caregiver Burden, Financial Strain, and Access to Care

When a pet is diagnosed with a chronic or terminal illness (e.g., cancer, diabetes, chronic kidney disease, heart failure), the owner’s role shifts from companion to caregiver, a transition that carries substantial psychological costs. A matched cross-sectional study of 119 owners of sick pets compared with 119 owners of healthy pets found that caregivers of sick animals reported significantly greater caregiver burden, perceived stress, depression, anxiety, and poorer quality of life [24]. Subsequent work has replicated these findings across cancer, diabetes, and chronic kidney disease [25,26]. A validated pet-specific caregiver burden scale—the 7-item abbreviated Zarit Burden Interview adapted for pets—has been developed [26]. Higher burden correlates with poorer psychosocial function and greater use of non-billable veterinary services (e.g., telephone calls, emails), which may reflect increased demands on veterinary staff [25].

The substantial lifetime costs of pet ownership—routine food, preventive care, and emergency treatment—may impose a financial strain that is often overlooked. Financial limitations are the most frequently reported barrier to accessing veterinary care [27]. Owners facing economic hardship may delay or forgo veterinary care, leading to guilt, anxiety, and poorer health outcomes for both the owner and the animal [27]. Access to veterinary care varies geographically and socioeconomically; barriers such as geographic location and limited availability of personnel or equipment are consistently identified [27]. Poor communication or lack of empathy from veterinarians, particularly around euthanasia decisions, can intensify owner guilt and complicate the grieving process [28,29]. Owners of very ill pets experience distress comparable to that documented in human caregiving literature [24], yet this burden remains underrecognized in both veterinary and human health settings [27,29].

### 3.4. Cognitive Dysfunction Syndrome: Relational Erosion in Aging Pets

Cognitive dysfunction syndrome (CDS), a progressive neurodegenerative disorder of aged dogs and cats analogous to early Alzheimer’s disease, is characterised by DISHAA: disorientation, altered interactions, sleep–wake cycle changes, house-soiling, activity changes, and anxiety [30]. Prevalence rises markedly with age: approximately 28% of dogs aged 11–12 years show at least one sign, rising to nearly 68% at 15–16 years [31]. Caring for a pet with CDS imposes a distinct burden. A survey of 516 dog owners compared caregivers of healthy ageing dogs, dogs with cancer, and dogs with canine cognitive dysfunction (CCD). CCD caregivers reported significantly greater time burden (due to nocturnal restlessness and disorientation), emotional distress (such as feelings of stigmatization), financial burden, and lower confidence in performing positive care tasks compared to the other groups [31]. Behavioural disruptions resemble those seen in human dementia caregiving, and emotional impacts include anticipatory grief [32], loss of the pet’s former identity, and sometimes resentment or guilt over negative feelings toward an animal that no longer seems recognizable [31]. CDS is not only a neurological disease but also a relational disease. A dog that once greeted its owner enthusiastically may become withdrawn or aggressive [33]; a reliably house-trained cat may begin eliminating inappropriately.

### 3.5. Pet Loss: Death, Euthanasia, Relinquishment, and Prolonged Grief

Pet death, whether natural, accidental, or by euthanasia, is an inevitable and potentially devastating event. In a recent study, approximately 7.5% of bereaved pet owners met clinical criteria for prolonged grief disorder [34]. The circumstances surrounding euthanasia have a substantial impact: owners who feel excluded from the decision or who perceive the timing as too early or too late report more intense grief, guilt, and complicated bereavement [35].

Relinquishment—the surrender of a pet due to behavioural problems, financial hardship, housing changes, or owner illness—is another form of loss that carries profound psychological consequences [20]. Owners who relinquish pets experience cognitive dissonance, psychological distress, and intense guilt, shame, and embarrassment. Pet relinquishment is associated with significant psychological distress, including depression and anxiety [20]. Unlike death, relinquishment is often an actively chosen loss, yet its psychological toll can be as severe as that of death. These forms of loss are often overlooked in the literature on pet health and human–animal interaction, but are critical to understanding the full range of emotional outcomes associated with pet ownership.

### 3.6. Zoonotic Risk and Owner Health Anxiety

**Figure 1:**
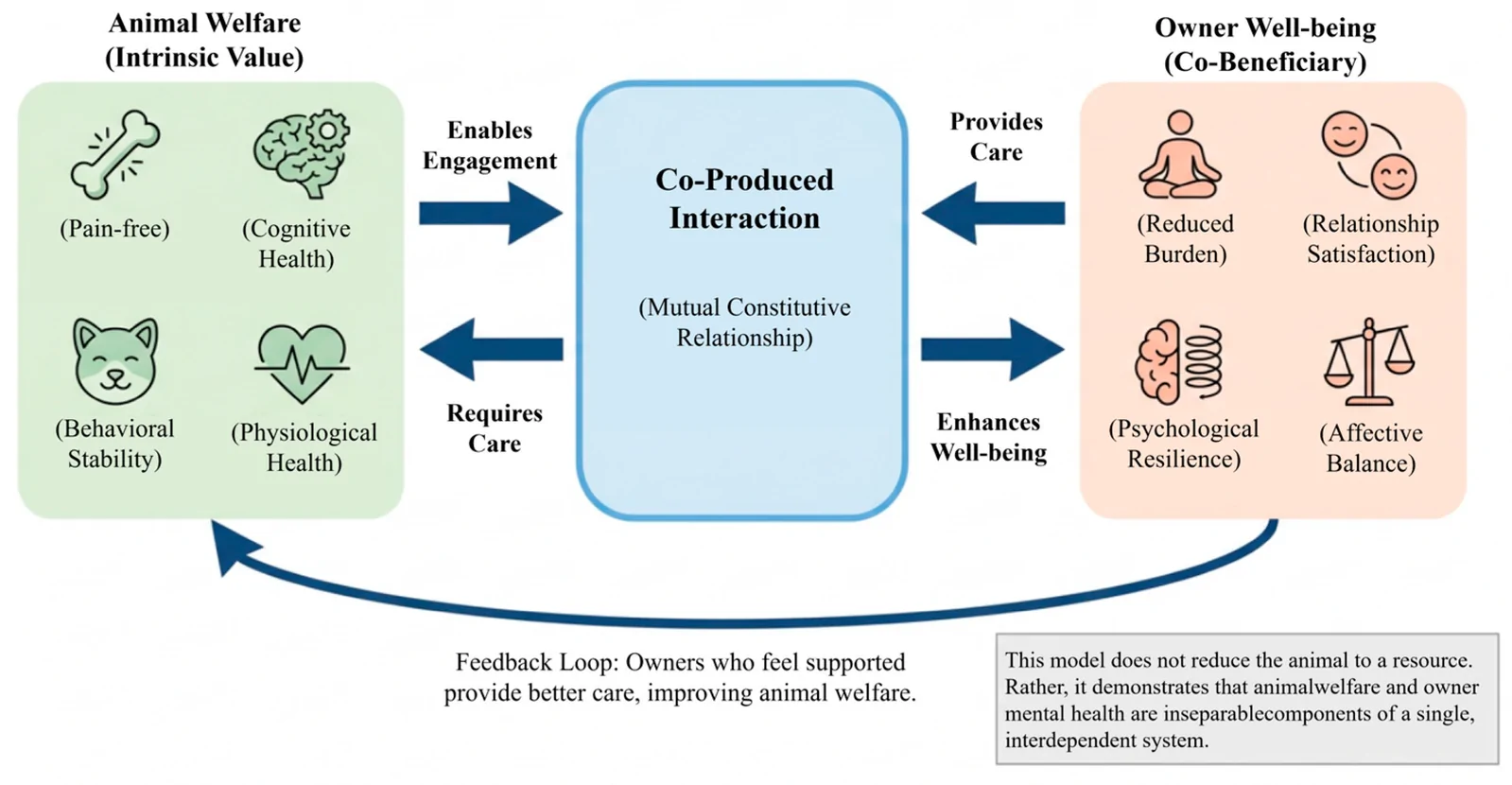
A Co-Produced Model of Human–Animal Wellbeing. This framework depicts the dynamic, interdependent relationship between animal welfare and owner well-being. On the left, animal welfare (Intrinsic Value) is represented by pain-free status, cognitive health, behavioural stability, and physiological health. On the right, owner well-being (Co-Beneficiary) is represented by reduced burden, relationship satisfaction, psychological resilience, and affective balance. The central element, “Co-Produced Interaction” (Mutual Constitutive Relationship), highlights that neither party is subordinate to the other; the relationship is co-produced. The bidirectional arrows illustrate the specific pathways: animal welfare enables engagement, which requires care from the owner; in turn, owner care provides for the animal and enhances the owner’s own well-being. The bottom feedback loop illustrates the cycle where owners who feel supported provide better care, thereby improving animal welfare. This model does not reduce the animal to a resource; rather, it demonstrates that animal welfare and owner mental health are inseparable components of a single, interdependent system.

Zoonotic diseases—such as toxoplasmosis, ringworm, flea- and tick-borne infections, and carriage of multidrug-resistant organisms (e.g., ESBL-producing *E. coli*)—pose a real, albeit generally low, risk to owners. For immunocompromised individuals (e.g., those undergoing chemotherapy, organ transplant recipients, or individuals with autoimmune diseases), this risk can become a source of chronic health anxiety that directly limits the quality and frequency of pet interaction [36,37]. Some owners may reduce physical contact, avoid certain activities (e.g., grooming, cleaning litter boxes), or even consider relinquishment [38]. While the evidence directly linking zoonotic risk perception to reduced relational Value is limited, clinical experience and qualitative reports suggest that fear of disease transmission can undermine the human–animal bond that pet ownership is intended to foster [39]. Veterinary guidance on safe handling practices may be essential to mitigate this anxiety without unnecessarily restricting positive interaction [40]. Figure 1 illustrates the co-produced model of human–animal wellbeing. It maps core dimensions of animal welfare (pain-free status, cognitive health, behavioural stability, and physiological health) onto owner well-being, demonstrating how the degradation of these welfare dimensions disrupts the bidirectional feedback loop that sustains both members of the dyad.

## 4. Nutritional Modulation of Pet Behaviour and the Quality of Human–Animal Interaction

This section synthesizes the empirical evidence on specific nutritional interventions. The primary objective of these interventions is to directly improve the animal’s physical and psychological welfare—by alleviating pain, reducing anxiety, and supporting cognitive function. This direct benefit to the animal is the central ethical justification for their use. Because the bond is co-produced, these improvements have a natural, secondary effect: they restore the animal’s capacity for positive engagement, which in turn reduces the owner’s burden and allows for more fulfilling interactions. However, the owner-outcome is not the primary target; it is an expected correlate of the animal’s improved quality of life.

### 4.1. Amino Acid Precursors: From Neurotransmitter Balance to Responsive Handling

The balance of excitatory and inhibitory neurotransmission in the central nervous system, particularly serotonergic and catecholaminergic tone, is directly influenced by the dietary supply of amino acid precursors. Tryptophan (Trp), the sole precursor of serotonin, is the best-studied example. Low serotonergic activity is associated with increased impulsivity, aggression, and poor stress coping in both humans and animals. In dogs, controlled supplementation with Trp has been shown to reduce owner-reported aggression, fearfulness, and reactivity to handling [41,42]. A systematic review concluded that there is moderate evidence for dietary Trp reducing anxiety-related behaviour [43], and a more recent evidence summary supports this conclusion while noting that Trp alone may not suffice as a sole treatment [44]. However, the existing evidence is derived primarily from small, short-term studies relying on owner-reported outcomes, and larger, blinded, placebo-controlled trials with standardized dosing and objective behavioural measures are needed to confirm these findings.

These behavioural changes may serve as a supportive adjunct to structured behaviour modification, potentially making daily interactions safer and more predictable. A dog that no longer snaps when its collar is grasped or when a child approaches its food bowl can remain a trusted family member. Daily routines—grooming, leash attachment, administering medication, gentle restraint—become low-stress events for both parties. Conversely, acute tryptophan depletion (relative to other large neutral amino acids) has been shown to exacerbate irritability and defensive aggression, and to reduce the perceived intimacy and romance in others’ close relationships [45,46]. Tyrosine (Tyr), the precursor of catecholamines, has received less attention in companion animals; however, evidence from other mammals suggests that tyrosine supplementation may help maintain cognitive and behavioural performance under stressful conditions [47], which could plausibly assist a pet in remaining cooperative during veterinary visits or travel, thereby reducing owner distress in high-anxiety situations.

### 4.2. Omega-3 Fatty Acids: From Pain Reduction to Restored Affectionate Contact

Dietary omega-3 polyunsaturated fatty acids (EPA and DHA) alleviate pain through anti-inflammatory mechanisms and improved joint comfort, as demonstrated by their ability to reduce pro-inflammatory cytokines and cartilage-degrading enzymes while promoting the production of specialised pro-resolving mediators [48]. In dogs with osteoarthritis, high-dose EPA (typically 50–100 mg/kg daily) consistently improves owner-assessed pain, mobility, and quality of life across multiple randomised controlled trials [49,50,51]. A rigorous systematic review and meta-analysis further concluded that omega-3-enriched diets and omega-3 supplements have evident clinical analgesic efficacy for canine osteoarthritis, whereas glucosamine-chondroitin products show a marked non-effect [52]. By reducing pain-induced behavioural withdrawal, omega-3 supplementation may serve as a valuable adjunct to appropriate pain management (e.g., NSAIDs) and physical rehabilitation, helping to restore the pet’s tolerance for gentle handling, willingness to engage in shared activities, and overall responsiveness to owner interaction—thereby potentially preserving the relational Value of the human–animal bond.

The behavioural signature of pain relief may be a pet that again tolerates gentle stroking, rises willingly for a walk, and initiates play—interactions that owners experience as deeply rewarding. When a previously withdrawn dog begins to nudge its owner’s hand for a belly rub or eagerly follows the lead for a morning walk, the relational Value of the relationship may be visibly restored. Omega-3 supplementation thus may act not only on the pet’s physical welfare but also on the relational dynamics that determine owner satisfaction [53]. In cats, the evidence is less extensive, but consistent findings support the benefits of omega-3s. Earlier studies also reported improved mobility and owner-assessed outcomes in arthritic cats [54,55]. A more recent 13-week randomised blinded study in 30 cats with naturally occurring OA demonstrated that a diet enriched with EPA/DHA significantly improved weight-bearing peak vertical force, stair performance, nighttime actimetry, and reduced functional decline, with the earliest signs of therapeutic response emerging at week 6 [56]. Collectively, these feline studies indicate that omega-3 supplementation can effectively manage OA-related pain and disability, with early intervention potentially yielding more rapid benefits. While multiple randomised controlled trials support the efficacy of omega-3 supplementation for canine osteoarthritis, most rely on owner-assessed outcomes, and the heterogeneity in dosing regimens (ranging from 50 to 100 mg/kg EPA) and outcome measures limits direct comparability across studies.

### 4.3. The Gut–Brain Axis: From Anxiety-Driven Problem Behaviours to a Calmer Household

As reviewed earlier, problem behaviours such as aggression, separation distress, and destruction are major predictors of owner frustration and relinquishment [20]. Crucially, these behaviours are primarily driven by environmental and management factors, such as inadequate exercise, prolonged periods of isolation, and lack of environmental enrichment. Addressing these core deficits in the animal’s daily life (e.g., increasing exercise duration, reducing the time left alone, providing interactive toys and training) must be the first-line and primary intervention. Only after these fundamental behavioural and environmental needs are adequately met should nutritional strategies, such as those targeting the gut–brain axis, be considered as adjunctive components. Psychobiotics, live microorganisms that confer mental health benefits through gut–brain interactions, have shown promise in companion animals [57,58]. Emerging evidence links specific gut bacteria, such as Blautia, to anxiety scores in pet dogs [59], and dietary strategies including probiotics and prebiotics have been shown to alleviate stress-related outcomes [60].

A randomised, placebo-controlled trial of *Lactiplantibacillus plantarum* LP815TM in 40 home-based dogs found that dogs receiving the probiotic daily for four weeks showed significant improvements in aggression and anxiety compared with placebo controls [61]. These findings were corroborated by objective activity monitoring, which demonstrated faster post-departure settling, meaning the dog calmed more quickly after the owner left the house, reduced daytime sleep, and improved sleep consistency in the treatment group. Notably, similar anxiolytic effects have been reported with *L. plantarum* PS128 in dogs with aggression and separation anxiety [62], suggesting that GABA-producing *L. plantarum* strains may act through shared gut–brain mechanisms. It is important to note, however, that these probiotic interventions were evaluated in dogs maintained under adequate husbandry conditions. The evidence for psychobiotics in dogs is promising but currently limited to a small number of randomised controlled trials with relatively small sample sizes. Most studies rely on owner-reported behavioural assessments, which may be subject to placebo effects and observer bias. Furthermore, the specific strains, doses, and durations of treatment vary widely across studies, making it difficult to generalize findings. Independent replication studies are urgently needed. These changes may translate into a home environment with less conflict, less property damage, and fewer thoughts of relinquishment, thus potentially preserving the human–animal bond. Moreover, the use of probiotics carries none of the sedation or side effects associated with psychopharmacological agents, making them suitable for long-term use only as part of a comprehensive behavioural plan. In multiple pet households, reduced inter-animal aggression may also improve the owner’s ability to manage the whole group, increasing the time spent in positive, multi-pet interactions.

### 4.4. Medium-Chain Triglycerides: Preserving Interactive Capacity in Aging Pets

As described earlier, CDS progressively impairs a pet’s ability to recognise its owner and engage in previously enjoyed interactions, often leading to owner distress and withdrawal. Nutritional strategies that slow cognitive decline, therefore, may play a supportive role in preserving the emotional bond in aging pets, when integrated with environmental enrichment, cognitive stimulation, and regular veterinary monitoring.

Medium-chain triglycerides (MCTs) have emerged as the most robustly supported nutritional intervention for CDS [63,64]. Ageing brains exhibit reduced cerebral glucose metabolism; MCTs are metabolised to ketone bodies, which can serve as an alternative energy substrate for glucose-impaired neurons. In a double-blind, placebo-controlled study, aged Beagle dogs maintained on a diet supplemented with 5.5% MCT for eight months showed significantly better performance on a battery of cognitive tests than control dogs, with larger effects observed on more difficult tasks [65]. Additionally, a randomised controlled trial of a commercial diet containing MCTs, antioxidants, and omega-3 fatty acids demonstrated significant improvements in owner-rated clinical signs of CDS, including disorientation, altered social interactions, and house-soiling [30]. Beyond cognitive enhancement, MCT supplementation has been shown to improve mitochondrial function and reduce steady-state amyloid precursor protein levels in the aged dog brain, suggesting neuroprotective effects that may slow disease progression [66].

The behavioural gains, such as a dog that again greets its owner at the door, may directly restore the moments of affectionate contact that define the relational Value of pet ownership. Even partial improvement could potentially transform the owner’s caregiving experience from a source of anticipatory grief into an extension of meaningful companionship. Conversely, untreated CDS leads to progressive withdrawal and relational rupture; nutritional cognitive support is therefore a relational intervention as much as a neurological one. As with the interventions discussed above, the evidence for MCT supplementation is limited by small sample sizes, short durations, and a lack of long-term follow-up data (see Section 6 for a comprehensive assessment of evidence quality).

### 4.5. Functional Ingredients That Target Specific Barriers to Positive Interaction

Beyond the major pathways described above, several functional ingredients have been developed as adjunctive tools to address specific behavioural barriers that directly obstruct positive human–animal contact.

Alpha-casozepine, a milk-derived decapeptide obtained from tryptic hydrolysis of bovine αs1-casein, exhibits affinity for the GABAA receptor and exerts benzodiazepine-like anxiolytic effects without sedation or tolerance [67,68]. Multiple studies have shown that alpha-casozepine reduces fearfulness and anxiety in dogs during veterinary visits, thunderstorms, and travel [69,70]. A recent randomised, fully blinded, placebo-controlled crossover study demonstrated that a six-day administration of alpha-casozepine significantly lowered low body postures, lowered ears, lowered tail, and lowered head, while increasing olfactory exploration during a standardised veterinary examination [70]. A dog that remains calm may accept petting and complete veterinary procedures without stress, potentially turning a traumatic event into a manageable routine. This means that a veterinary visit, rather than being a traumatic event that erodes trust, can become a manageable routine. Similarly, a dog that tolerates car travel allows the owner to include the pet in holidays and outings, increasing shared positive experiences.

L-theanine, a tea-derived amino acid (N-ethyl-L-glutamine), increases brain concentrations of gamma-aminobutyric acid (GABA), serotonin, and dopamine, thereby inhibiting excitatory neurotransmission [71]. In a laboratory model of anxiety-related behaviour, L-theanine (25–50 mg twice daily) significantly reduced fearful behaviour toward unfamiliar human beings, as evidenced by longer duration of interaction and time spent near the human, without any motor stimulant or sedative effects [72]. For the owner, this may directly improve the ability to introduce the dog to guests, walk confidently in public spaces, and allow trusted friends to pet-sit without incident. These effects are not merely cosmetic: they reduce the owner’s chronic vigilance and embarrassment, allowing spontaneous, relaxed interaction that would otherwise be impossible. Similarly, the evidence for alpha-casozepine and L-theanine is limited by small sample sizes, short durations, and reliance on owner-reported or observer-rated measures (see Section 6 for a comprehensive assessment of evidence quality).

Evidence grading criteria for Table 1: To ensure transparency in our evidence-strength classifications, we applied the following pre-specified criteria. Strong evidence denotes support from ≥3 independent randomised controlled trials (RCTs) with adequate power, blinding, validated outcome measures (including objective assessments where feasible), and consistent findings across studies. Moderate evidence denotes support from 1–2 RCTs, or from multiple studies with methodological limitations (e.g., small sample sizes, reliance on owner-reported outcomes, short intervention durations, or lack of blinding), or when findings were not entirely consistent. Preliminary evidence denotes support solely from uncontrolled observational studies, case reports, or conflicting findings that precluded definitive conclusions.

## 5. A Structurally Embedded Framework for Co-Produced Pet-Owner Outcomes

This section presents our co-produced framework, which is fundamentally embedded within material and social conditions. We argue that financial hardship, housing instability, and limited access to veterinary care are not peripheral context, but the primary structural determinants that shape the caregiving capacity of the owner and the welfare of the animal. The framework explicitly rejects the psychologization of structural constraint: an owner who cannot afford analgesia or lives in housing that precludes daily exercise should not be modelled as simply ‘insecurely attached’ or ‘inconsistent’. Instead, these material constraints constitute the foundational layer of the dyad, and psychological factors (attachment style, anxiety) operate within, and are often downstream of, these structural realities.

### 5.1. Structural Constraints and Owner Psychology Jointly Shape Pet Welfare

To understand how owners affect pet physiology and behaviour, we must first establish the primacy of structural determinants. Housing insecurity, unemployment, and food poverty directly limit an owner’s ability to provide consistent veterinary care, quality nutrition, and daily exercise. These material deprivations are proposed as upstream antecedents for pet health deterioration. Only after establishing this foundation can we consider psychological factors. While it is true that affiliation with pets can elevate oxytocin and reduce cortisol [73,74,75], these positive interactions are only possible when the owner has the material resources to support them.

Once structural constraints are acknowledged, the owner’s psychological state is conceptualized as a secondary contributing factor that may amplify existing vulnerabilities; however, establishing causal primacy requires experimental designs. Correlational evidence shows that owner trait anxiety is associated with dog behaviour problems; for example, long-term stress levels have been shown to be synchronized between dogs and their owners [76]; however, this anxiety may arise as a downstream consequence of the financial and environmental stressors the owner faces. Potential, but largely untested, mechanisms underlying these correlations include emotional contagion and reduced environmental enrichment [77]. Although these pathways are biologically plausible, they currently rest on correlational evidence and require mechanistic validation. They are also likely to operate within, and frequently downstream of, the material realities described above. Similarly, in cats, owner neuroticism has been linked to poorer welfare [78]; however, these associations may partly reflect the owner’s reduced capacity to provide enrichment under constrained conditions, rather than a purely psychological origin. Therefore, assessing the owner’s material environment must precede any evaluation of their psychological state.

Importantly, owner attachment style moderates these dynamics, alongside socioeconomic status, household composition, previous psychiatric history, personality traits, and life stressors. Insecurely attached owners, particularly those with high anxiety, may display hypervigilant, intrusive caregiving that paradoxically increases pet stress, whereas avoidantly attached owners may provide minimal interaction, depriving pets of positive social input. Both patterns can trigger or exacerbate behavioural problems, creating a self-perpetuating cycle [79].

### 5.2. Feeding Practices as a Pathway Where Material Constraints and Owner Psychology Intersect

Feeding practices are shaped by both material constraints and psychological factors. For low-income households, food is often the most affordable tool for providing affection and comfort, making it a materially constrained expression of care rather than merely a symptom of ‘emotional feeding’. A cross-sectional study of 563 dog and cat owners found that owners with higher attachment anxiety gave more treats per day and had pets (particularly dogs) with larger body condition scores [80]. This finding is consistent with our framework: anxious owners may engage in more solicitude caregiving, but the availability of affordable food as a primary reward currency is itself a structural constraint. Conversely, avoidant attachment was associated with fewer treats and less daily interaction, which may reduce obesity risk but also deprive pets of positive engagement. However, this pattern may also reflect an owner’s limited financial capacity to purchase treats or time constraints due to work obligations, rather than purely a psychological origin.

Treats may serve functions extending well beyond nutrition. Although a qualitative study identified their roles in behaviour management and medication delivery [81], treat-giving may also provide owners with immediate gratification and reinforce perceived affection. Crucially, for owners facing financial hardship, treats may represent the only affordable avenue for providing positive reinforcement and comfort to their pets. Effective interventions must therefore provide affordable, non-food alternatives (e.g., play, grooming, training) and structured guidance on treat types and quantities that minimise caloric impact while preserving relational function [82]. Furthermore, socioeconomic constraints, cultural norms, and household composition must be treated as first-order determinants of feeding practices, not merely additional moderators of attachment style.

### 5.3. Vicious and Virtuous Cycles: Implications for Breaking the Loop

The bidirectional pathways described above interact to produce dynamic cycles. In a virtuous cycle, a securely attached owner provides consistent, predictable, and affectionate care, including appropriate nutrition and positive interaction. The pet, receiving these inputs, experiences low stress, good behavioural regulation, and robust physical health. A healthy, thriving pet experiences a higher quality of life and a reduced burden of suffering. This thriving state, in turn, allows for more positive and reciprocal interactions, reinforcing the owner’s capacity to provide high-quality care within a mutually beneficial cycle [79,83].

In a vicious cycle, the entry point is often structural rather than psychological. Financial hardship or housing instability directly prevents an owner from accessing veterinary care or providing therapeutic nutrition, leading to the pet developing severe health problems (e.g., unmanaged pain, obesity, or anxiety). These pet-level health deteriorations subsequently generate profound psychological distress in the owner (burden, guilt, depression), which is a downstream consequence of the material failure, not the root cause. The pet’s deteriorating condition further exacerbates owner stress, frustration, and caregiver burden, confirming anxious expectations and deepening the cycle [20,24]. Therefore, within our framework, we hypothesize that unless structural access barriers are addressed, psychological or nutritional interventions alone may have limited effectiveness in breaking the cycle. This proposition requires rigorous testing before it can be confirmed.

**Figure 2:**
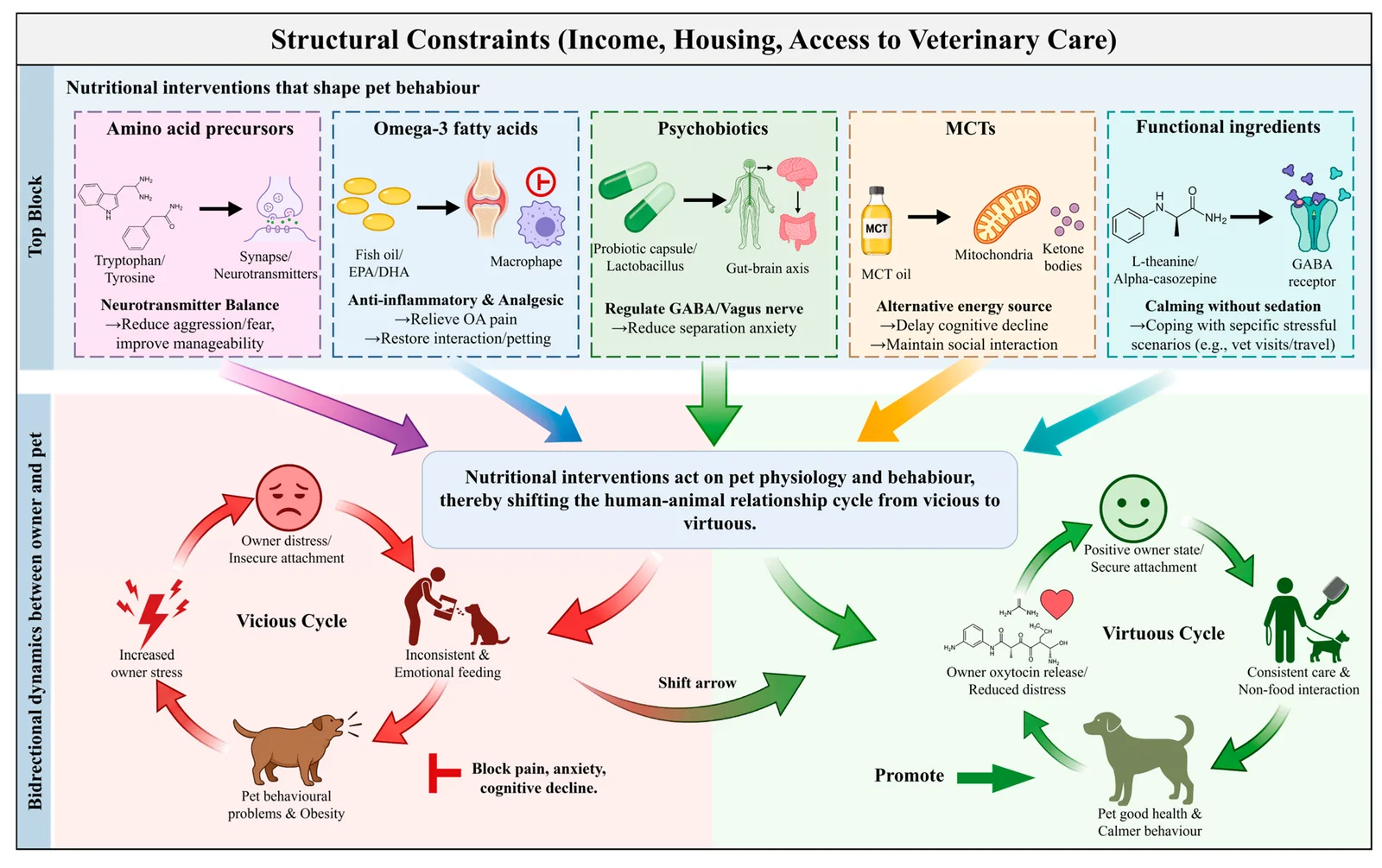
A proposed conceptual model of the bidirectional loop of human–animal interactions, embedded within structural constraints. This framework illustrates the reciprocal feedback between nutritional interventions, pet behaviour, and owner psychology. The overarching context at the top indicates that the entire cycle operates within material and social conditions—including financial hardship, housing instability, and limited access to veterinary care. In this model, these structural constraints are proposed as key determinants of both the owner’s capacity to provide care and the pet’s welfare, meaning that an owner who cannot afford analgesia or lives in housing that precludes exercise faces fundamentally different constraints than an owner with adequate resources. Therefore, we hypothesize that without alleviating these structural barriers, nutritional interventions alone cannot successfully shift the cycle from vicious to virtuous. The six nutritional interventions (amino acid precursors, omega-3 fatty acids, psychobiotics, MCTs, alpha-casozepine, and L-theanine) target neurobiological pathways that are hypothesized to reshape pet behaviour. In turn, these changes may improve interaction quality and owner well-being, thereby sustaining a virtuous cycle—provided that the surrounding structural constraints are simultaneously alleviated. Note: The pathways depicted in this figure represent a conceptual model derived from existing correlational evidence. The specific claim that nutritional interventions lead to measurable improvements in owner well-being through changes in pet behaviour has not been directly tested in randomized controlled trials and should be regarded as a testable hypothesis, not a demonstrated outcome.

Breaking these cycles may require dyadic interventions that target both members simultaneously. Three entry points are particularly promising in this regard. First, owner psychological support: addressing anxiety, depression, or attachment insecurity can directly improve caregiving consistency and reduce emotional feeding [24,79]. Second, environmental and behavioural restructuring: this is the most critical and foundational step. It involves modifying the animal’s living conditions, such as ensuring adequate daily exercise, providing enrichment toys, reducing the duration of isolation, and implementing consistent training routines. Nutritional interventions should be considered a supportive third step in this hierarchy. As reviewed earlier, targeted nutrition can reduce pain, improve behaviour, and support cognitive function, but only when integrated into these core environmental and behavioural changes [53,84]. Third, feeding practice modification: replacing food-based affection with non-food alternatives, using interactive feeders, and providing owner education on treat composition and portion control can reduce obesity risk while preserving the relational function of feeding [81,82,85]. The key insight is that interventions focused solely on the pet (e.g., a therapeutic diet) or solely on the owner (e.g., mental health counselling) are unlikely to achieve durable change; rather, effective strategies must address the relationship itself. Despite the logical appeal of dyadic interventions, no randomized controlled trial has yet tested whether combining owner psychological support with pet nutritional intervention produces greater benefits than either alone—a critical gap for future research. Figure 2 illustrates the bidirectional cycles through which nutritional interventions and owner psychology influence each other, mediated by changes in pet behaviour and daily interaction quality.

## 6. Translational Outlook: From Mechanistic Insights to Evidence-Based Interventions

Before discussing the translational implications of the evidence reviewed above, it is important to critically assess the overall quality of the evidence base. Most studies in this field are small (typically involving fewer than 100 animals), short-term (lasting 4–12 weeks), and rely primarily on owner-reported outcome measures rather than objective assessments. Blinding and control groups are not consistently implemented, and the heterogeneity of outcome measures limits comparability across studies. Furthermore, publication bias may favour positive findings, and negative results are less likely to be reported. These limitations should be kept in mind when interpreting the conclusions of this review. The translational implications and research agenda presented in this section are derived from the conceptual framework proposed in Section 5. The recommendations made here are forward-looking and should be understood as priorities for future research rather than established clinical guidelines. Building on this evidence base and the proposed framework, this section synthesises the major research gaps that currently limit evidence-based translation, proposes methodological priorities for future studies, and outlines practical pathways for integrating nutritional strategies into veterinary practice, pet food innovation and one health collaborations.

### 6.1. The Owner-Outcome Gap: A Call for Dyadic Endpoints

It is important to note that the bidirectional framework underpinning the translational recommendations in this section is a conceptual model. While the direct benefits of the nutritional interventions reviewed in Section 4 on pet health and behaviour are supported by empirical evidence, the proposed downstream effects on owner well-being and the human–animal bond remain theoretical. The translational implications discussed below should therefore be interpreted as priorities for future research, rather than established clinical or commercial guidelines.

Nutritional interventions can change pet behaviour, but it remains almost entirely untested whether those changes actually improve the owner’s emotional well-being, relationship satisfaction, or perceived relational Value. That missing link is the most critical limitation of the current literature. Recommended design shift: Future randomised controlled trials should adopt a co-equal dyadic design, where the animal’s quality of life, pain scores, and behavioural welfare are treated as equally important primary endpoints as the owner’s emotional well-being. Neither outcome should be considered a mere proxy for the other; both are intrinsically valuable targets of intervention. For the pet: validated behaviour scales such as the Canine Behavioural Assessment and Research Questionnaire (C-BARQ) or its shortened version [86,87], and where feasible, objective measures (activity monitors, salivary cortisol, oxytocin). For the owner: validated instruments including the Lexington Attachment to Pets Scale (LAPS) or its brief version [88,89], the Dog Owner Relationship Scale (DORS28/DORS12) as a measure of relationship quality [90], validated caregiver burden scales, and standard mental health measures such as the Patient Health Questionnaire-9, Generalized Anxiety Disorder-7, and Perceived Stress Scale. Where resources permit, ecological momentary assessment (daily diaries of interaction quality, conflict events, moments of affectionate contact) can capture real-world dynamics that retrospective questionnaires miss, as demonstrated by recent experience sampling studies [91].

### 6.2. Standardising Behavioural and Nutritional Assessment in Companion Animals

A second major obstacle to synthesis and meta-analysis is the marked heterogeneity in behavioural assessment tools, nutritional intervention protocols, and outcome timeframes across studies [92]. Some trials rely on owner-reported global impressions of change, others use standardised behaviour inventories such as the C-BARQ or the Canine Cognitive Dysfunction Rating Scale (CCDR), and still others depend on veterinary clinical judgment [93,94,95,96]. The dose, duration, and formulation of nutritional ingredients vary widely, making cross-study comparison difficult [47,97]. To address these issues, the field would benefit from a consensus-based core outcome set for pet behaviour trials, analogous to initiatives in human medicine such as the COMET initiative. At a minimum, studies should report validated, published instruments and provide raw data or effect sizes for key behavioural domains, including aggression, separation-related distress, fear, and cognitive function [93,98].

For nutritional interventions, minimum reporting standards are urgently needed. Authors should clearly specify ingredient source, purity, dose (expressed as mg/kg body weight or percentage of metabolisable energy), duration of intervention, and timing of outcome assessment. Blinding of owners and outcome assessors should be standard practice to reduce bias [99,100,101]. The adoption of standardised behavioural challenge protocols could further improve comparability across studies. For situational anxiety triggered by events such as veterinary visits, travel, or noise exposure, field-based standardised challenges—for example, a simulated veterinary examination or playback of recorded thunderstorm sounds—have proven useful in eliciting and quantifying fear-related behaviours [102,103,104]. Collectively, these measures would enhance the reproducibility, comparability, and overall quality of research in companion animal behaviour and nutrition. Additionally, future studies should prioritise: (i) the inclusion of objective outcome measures (e.g., activity monitors, salivary cortisol, actigraphy) alongside owner-reported instruments; (ii) adequate sample sizes with power calculations; (iii) blinding of owners and outcome assessors; and (iv) reporting of effect sizes and confidence intervals to facilitate meta-analysis. These methodological improvements are essential for advancing the evidence base from preliminary observations to robust, actionable conclusions.

### 6.3. From Short-Term Efficacy to Long-Term Effectiveness and Dose–Response

Most nutritional intervention studies in companion animals remain short-term, typically lasting 4 to 12 weeks [30,105]. Consequently, it is unclear whether behavioural benefits persist with continued use, require ongoing administration, or produce lasting changes that outlast the intervention. A 12-month longitudinal feeding study in dogs with IRIS-Stage 1 chronic kidney disease demonstrated sustained improvement in renal biomarkers and quality of life [106], but long-term behavioural data are scarce. In a 126-day pilot trial combining omega-3 fatty acids, magnesium, and zinc, improvements in fearfulness and destructiveness were observed during the 6-week treatment period, yet inappropriate elimination rebounded after treatment cessation, suggesting that withdrawal effects may occur [107]. Only one study examined post-intervention durability, reporting that cognitive gains returned to baseline within 10 days after stopping supplementation [84,108]. Thus, longer-term trials with multiple assessment points and a post-intervention follow-up phase are urgently needed.

Dose–response relationships are also poorly characterised. In a graded Trp supplementation study (0.05–0.15% added on top of 0.18% basal), no consistent behavioural improvement was observed in healthy hounds, possibly because the achieved Trp:large neutral amino acid ratio remained below the proposed 0.061:1 threshold [109]. Conversely, a crossover trial in aggressive dogs found that Trp-supplemented low-protein diets (Trp:LNAA ≈ 0.07:1) reduced dominance aggression, while high-protein unsupplemented diets exacerbated it [41]. For omega-3 fatty acids, a systematic review concluded that higher doses of DHA (≥33 mg/kg) and EPA (≥27 mg/kg) consistently benefit cognition, whereas lower doses yield variable effects [85]. However, optimal doses may differ by breed, size, or baseline behavioural status, and dose-finding studies using randomised, blinded designs with at least three dose levels plus placebo remain rare. Finally, pharmacokinetic/pharmacodynamic studies in dogs and cats are almost entirely lacking; no published work has systematically linked nutrient absorption, brain penetration, and subsequent behavioural outcomes. Addressing these gaps is essential to move from empirical supplementation to evidence-based nutritional management of behaviour.

### 6.4. Mechanistic Biomarkers to Bridge the Preclinical-Clinical Gap

As discussed in the context of omega-3 fatty acids, MCTs and gut–brain signalling, plausible biological mechanisms have largely been proposed based on rodent studies or human extrapolation. Direct evidence that these same mechanisms operate similarly in dogs and cats remains often circumstantial [58,84]. The inclusion of validated mechanistic biomarkers in clinical trials would greatly strengthen causal inference and enable patient stratification, an approach increasingly recognised as essential for translating preclinical findings into practice [53,110].

Candidate biomarkers span several pathways. For the Trp/serotonin pathway, useful measures include the plasma Trp/large neutral amino acid ratio, the kynurenine/Trp ratio, and serum or platelet serotonin content; these have been linked to behavioural traits in dogs [111,112]. For omega-3 fatty acids, the red blood cell or plasma EPA + DHA percentage (omega-3 index) is a robust biomarker of long-term status, and plasma lipid mediator profiles (e.g., specialised pro-resolving mediators) can provide functional insight [113,114,115]. For the gut–brain axis, faecal short-chain fatty acids, shotgun metagenomic sequencing of faecal microbiome composition, serum lipopolysaccharide-binding protein as a marker of gut permeability, and salivary/urinary oxytocin as a downstream indicator of vagal activation have all shown promise [112,116,117]. For CDS and MCT interventions, plasma β-hydroxybutyrate confirms target engagement, serum brain-derived neurotrophic factor (BDNF) reflects neurotrophic support, and standardised cognitive testing batteries provide functional readouts [30,65,110]. When such biomarkers are measured, studies should report both baseline values and change scores, and explore whether baseline levels predict treatment response, thereby enabling a precision nutrition approach [53,84].

### 6.5. Integrating Nutritional Interventions into Dyadic Care Models

The bidirectional framework implies that effective care should address both the pet and the owner simultaneously, rather than treating each in isolation. Nutritional interventions are particularly well suited to this dyadic model, as they can enhance the pet’s biological capacity for calm, pain-free, and cognitively intact behaviour, which in turn may improve the owner’s emotional state and caregiving consistency. Given that canine anxiety- related traits such as fearfulness and noise sensitivity affect 29−32% of pet dogs [118], and that problem behaviours in dogs are associated with increased depression and caregiver burden in owners [21], we propose that integrating nutrition into dyadic care may be a highly relevant strategy. However, this proposition remains a plausible hypothesis, as direct evidence linking nutritional interventions to owner emotional outcomes has not yet been measured in controlled studies. As reviewed extensively in Section 4, specific nutritional interventions—such as omega- 3 fatty acids, magnesium, and zinc for fearfulness; probiotics like LP815TM for aggression and anxiety; and MCT- enriched diets for cognitive decline—have demonstrated potential to support pet welfare. Integrating these evidence- based strategies into dyadic care is therefore a logical next step.

Importantly, these care pathways should be understood as integrating nutrition into existing practice, not replacing standard veterinary or behavioural care. Three care pathways are proposed. First, environmental and lifestyle assessment should be the mandatory initial step. In veterinary behavioural practice, evaluating the animal’s daily schedule (e.g., hours left alone, physical exercise duration, access to enrichment) must precede any recommendation. Second, once these deficits are identified and corrected (e.g., implementing a structured exercise and training plan), nutritional assessment should follow as a routine adjunctive measure. In primary care and mental health settings, when a patient presents with depression, anxiety, or loneliness, clinicians can ask about pet ownership and offer simple, vet- overseen advice—such as managing the pet’s pain with omega- 3s, supporting an aging pet’s cognition with MCTs, or using a probiotic to reduce problem behaviours [119]. Finally, integrated one health clinics, where human healthcare providers, mental health professionals, and veterinarians collaborate, remain rare but highly promising; pilot studies with shared records and joint case discussions could generate proof- of- concept for wider implementation [120].

### 6.6. Industry Opportunities: Prioritizing Animal Welfare and the Co-Produced Bond

The pet food industry has a substantial opportunity to translate current evidence into products that prioritize animal welfare as an intrinsic goal. Improving the animal’s physical and psychological well-being may enrich the co-produced bond by reducing caregiver burden and restoring joyful, positive interactions. However, the direct effects on owner emotional well-being remain a plausible hypothesis; they have not been directly measured in controlled studies. We strongly advocate that any industry claims related to owner emotional outcomes must be substantiated by studies that include owner-reported emotional measures as co-primary endpoints. For stress management diets, formulations incorporating alpha-casozepine [69], specific probiotic strains such as *Lactiplantibacillus plantarum* PS128 [62], L-theanine, and tryptophan have shown potential for alleviating situational and chronic anxiety. However, rigorous randomised controlled trials remain essential for credible claims. For aging pets, cognitive support diets enriched with medium-chain triglycerides have demonstrated efficacy [30]. For pain management, high EPA omega-3 formulations are supported by multiple randomised controlled trials [49,51,56,121]. Such diets should be positioned explicitly as interventions that have demonstrated pet-level benefits and are hypothesized to help restore the pet’s willingness to engage in affectionate contact and shared activities, pending validation of owner-level outcomes. Critically, product claims related to emotional outcomes must be substantiated by studies that include owner-reported emotional measures, not merely pet behaviour scores. The industry has a unique opportunity to fund high-quality independent research that meets the standards of evidence expected in human healthcare.

### 6.7. Ethical and Practical Considerations for Implementation

Any translational effort must be grounded in animal welfare and equity. Nutritional interventions should never replace appropriate veterinary care, including pain management (e.g., NSAIDs for moderate to severe osteoarthritis) or behavioural modification under professional guidance. Nutraceutical use should be evidence-informed, not marketing-driven. Although some supplements show promise, study quality varies; enriched diet trials generally have higher methodological quality than supplement trials [84]. Even well-studied compounds like omega-3 fatty acids carry risks such as gastrointestinal upset or drug interactions [53]. Equity is equally critical: pet ownership and veterinary care access are strongly patterned by socioeconomic position. In a U.S. national sample, nearly 30% of pet-owning households fell into moderate or poor access groups, characterised by financial fragility and discrimination [122]. Underserved populations (low-income, unhoused, rural) may lack access to beneficial nutritional interventions. Beyond access to care, socioeconomic position and household composition should be recognized as key moderators of the relationships described in this review, as they may shape both owner well-being and pet management practices. Industry subsidies, sliding scale fees, or public health integration (e.g., “Peticaid”) warrant exploration [123].

Animal welfare demands safety transparency. Ingredients with psychoactive effects (e.g., alpha-casozepine, L-theanine) are generally safe, but long-term data are limited; plant extracts like garlic can cause haemolytic anaemia in dogs [53]. Transparency in formulation, independent safety testing, and pharmacovigilance should be standard practice, yet current adverse event reporting remains largely passive and industry self-regulated [53]. Without robust surveillance, there is a risk that nutritional interventions could compromise animal welfare; however, this remains a pragmatic concern rather than an empirically established causal pathway.

### 6.8. A Prioritised Research Agenda

**Figure 3:**
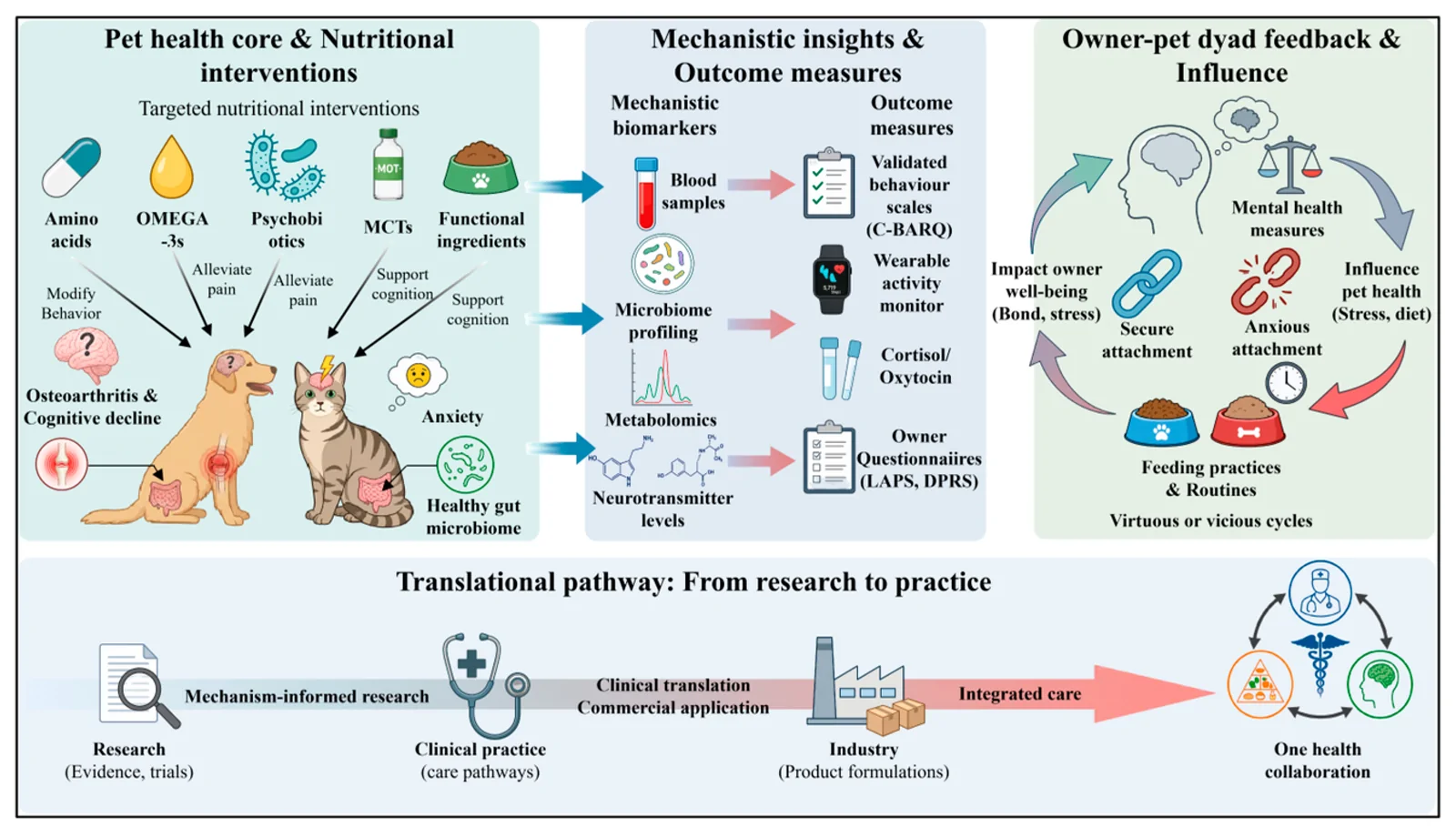
Translating mechanistic insights into evidence-based interventions in pet behavioural and nutritional health. This framework outlines the translational pathway from targeted nutritional interventions (amino acids, omega-3s, psychobiotics, MCTs, and functional ingredients) to mechanistic biomarkers and validated outcome measures, and highlights the bidirectional owner–pet dyad feedback loop, culminating in integrated pathways for research, clinical practice, industry formulation, and one health collaboration. Note: The downstream benefits to owner well-being depicted in the right-hand panel are plausible hypotheses derived from the proposed conceptual framework; they require dyadic, co-primary outcome trials to verify.

Based on the gaps identified above, the following research priorities are proposed in order of urgency and potential impact. First, dyadic randomised controlled trials that include co-primary outcomes for both pet (using validated behaviour scales such as C-BARQ or Fe-BARQ) and owner (assessing attachment, caregiver burden and mental health) are urgently needed. At least two such trials, one targeting anxiety or aggression (e.g., with probiotics or alpha-casozepine) and another focusing on cognitive dysfunction syndrome (e.g., with MCTs), would fundamentally transform the current evidence base [30,53,107]. Second, dose–response and long-term studies should be conducted for the most promising nutritional interventions, including omega-3 polyunsaturated fatty acids, MCTs, and psychobiotics, within multimodal treatment contexts that include standard behavioural modification and veterinary care, to establish minimum effective doses, optimal treatment durations, and durability of effects [124]. Third, mechanistic studies in target species (dogs and cats) using biomarker approaches such as metabolomics, microbiome profiling, and neurotransmitter analysis are required to confirm whether pathways inferred from rodent models operate similarly in companion animals [85,125]. Fourth, a core outcome set for pet behaviour trials should be developed and validated, ideally through an international Delphi process involving veterinarians, behaviourists, nutritionists, and owners. Existing validated instruments such as the C-BARQ and Fe-BARQ provide a strong foundation for this effort [93,126]. Fifth, pilot studies of integrated one health interventions that combine owner psychological support with pet nutritional management are recommended, measuring outcomes for both members of the human–animal dyad. Given that canine behavioural problems significantly increase owner burden and mental health symptoms, such integrated approaches hold promise for improving welfare on both sides of the leash [20]. Sixth, future research should explicitly collect and report data on potential confounding variables—including socioeconomic status, household composition, previous psychiatric disease, personality, attachment style, and life stressors—to enable robust statistical adjustment and to determine whether the observed relationships persist after controlling for these factors. Figure 3 outlines this roadmap. Collectively, these research priorities address critical gaps in the literature and offer a concrete, actionable framework for generating high-quality, clinically relevant evidence in companion animal behavioural nutrition.

## 7. Conclusions

The relational value of the human–animal bond is not an automatic consequence of pet ownership; it is a co-produced and dynamic outcome dependent on the animal’s intrinsic welfare and the owner’s psychological state. This review has advanced three interconnected propositions: that a pet’s own quality of life is fundamentally shaped by its health status across multiple domains; that targeted nutritional interventions play an adjunctive role in alleviating the animal’s direct suffering; and that the relationship is bidirectional, with owner psychological state feeding back to affect pet welfare. It is important to reiterate that our primary concern is the animal’s welfare as an end in itself, which, in a co-produced system, inevitably enriches the lives of both the animal and the owner. It is also important to reiterate that the propositions advanced in this review represent a synthesis of existing evidence combined with a proposed conceptual framework. We also note that the evidence reviewed here is heavily weighted toward canine studies; feline-specific data remain scarce, and conclusions should be applied to cats with caution. The first two propositions are supported by empirical evidence, while the third—the bidirectional model—is presented as a hypothesis-generating framework that requires future dyadic randomised controlled trials to verify.

The single most important gap is that owner emotional outcomes have almost never been measured. Closing this gap requires a deliberate shift from isolated, pet-centric trials to dyadic randomised controlled trials that include co-primary outcomes for both members of the dyad. Beyond this immediate methodological priority, the field must embrace four forward-looking challenges: first, moving from variable efficacy to precision dyadic stratification, identifying which owner–pet pairs benefit most from which nutritional strategy; second, extending short-term efficacy trials to real-world pragmatic effectiveness studies that assess durability, dose–response and cost-effectiveness; third, developing and validating mechanistic biomarkers (plasma Trp:LNAA ratio, omega-3 index, faecal short-chain fatty acids, serum BDNF, salivary oxytocin) to enable precision nutritional management; and fourth, integrating nutritional assessment into routine veterinary behavioural practice and piloting one health interventions that combine pet nutritional support with owner psychological care. What is now required is not more descriptive cross- sectional surveys or small uncontrolled trials, but a coordinated, interdisciplinary effort that treats the human- animal dyad as a single unit of analysis. To achieve this, a deliberate, funded, and collaborative agenda is essential—one that generates the high- quality evidence necessary to transform how we feed, care for, and value our companion animals, and, in doing so, improve the welfare of both pets and the households that care for them.

## Figures and Tables

**Table 1:** Summary of nutritional interventions, proposed mechanisms, strength of evidence, behavioural outcomes, and current evidence regarding owner outcomes.

Nutritional Intervention	Proposed Mechanism	Strength of Evidence	Behavioural Outcomes	Current Evidence on Owner Outcomes
Tryptophan	Serotonin precursor; modulates serotonergic neurotransmission [45,46]	Moderate (small, short-term studies; mostly owner-reported outcomes) [43,44]	Reduced owner-reported aggression, fearfulness, and reactivity to handling [41,42]	Not measured directly. Indirect evidence suggests that reduced aggression may improve manageability and reduce owner stress, but no study has measured owner emotional outcomes [41,42,43,44]
Omega-3 fatty acids (EPA/DHA)	Anti-inflammatory; reduces pro-inflammatory cytokines and cartilage-degrading enzymes; promotes pro-resolving mediators [48]	Strong (multiple RCTs in dogs with OA; fewer and less consistent in cats) [49,50,51,52,54,55,56]	Improved mobility, reduced pain-induced behavioural withdrawal, increased tolerance for gentle handling, restored willingness to engage in shared activities [49,50,51,54,55,56]	Not measured directly. Indirect evidence suggests that pain relief may restore affectionate contact and improve interaction quality, but no owner outcomes were assessed [49,50,51,52,53]
Probiotics/Psychobiotics	Gut–brain axis modulation; GABA production by specific strains (e.g., *L. plantarum*) [57,58,59,60]	Moderate (few small RCTs; owner-reported outcomes, some objective activity monitoring) [61,62]	Reduced aggression and anxiety, faster post-departure settling, reduced daytime sleep, improved sleep consistency [61,62]	Not measured directly. Indirect evidence suggests that reduced problem behaviours may alleviate owner burden, but no owner outcomes were measured [20,61,62]
Medium-chain triglycerides (MCTs)	Ketone bodies as alternative cerebral energy substrate for glucose-impaired neurons; improved mitochondrial function [63,64,66]	Moderate (limited number of double-blind RCTs; short duration, no long-term follow-up) [30,65]	Improved cognitive performance, reduced disorientation, improved social interactions, reduced house-soiling [30,65]	Not measured directly. Indirect evidence suggests that cognitive improvement may restore affectionate interaction and reduce caregiver strain, but no owner outcomes were measured [30,65]
Alpha-casozepine	GABAA receptor modulation; benzodiazepine-like anxiolytic effects without sedation or tolerance [67,68]	Moderate (small, short-term studies; laboratory and veterinary settings) [69,70]	Reduced fearfulness and anxiety during veterinary visits, thunderstorms, and travel; reduced low body postures, lowered ears/tail; increased olfactory exploration [69,70]	Not measured directly. No owner outcomes assessed in any identified study [69,70]
L-theanine	Increases GABA, serotonin, and dopamine concentrations; inhibits excitatory neurotransmission [71]	Moderate (laboratory-based studies; small sample sizes) [72]	Reduced fearful behaviour toward unfamiliar humans; longer duration of interaction and time spent near humans [72]	Not measured directly. No owner outcomes assessed in any identified study [72]

Note: The ‘Behavioural Outcomes’ column primarily represents direct improvements in the animal’s own welfare (e.g., pain relief, reduced anxiety), which is the primary target of these interventions. The owner outcomes column reflects the downstream relational correlates of these welfare improvements, not the primary endpoint.

## Data Availability

No new data were created or analyzed in this study. Data sharing is not applicable to this article.

## Abbreviations

The following abbreviations are used in this manuscript:

BDNF	Brain-Derived Neurotrophic Factor
C-BARQ	Canine Behavioural Assessment and Research Questionnaire
CCD	Canine Cognitive Dysfunction
CCDR	Canine Cognitive Dysfunction Rating Scale
CDS	Cognitive Dysfunction Syndrome
DHA	Docosahexaenoic Acid
DISHAA	Disorientation, altered Interactions, Sleep–wake cycle changes, House-soiling, Activity changes, and Anxiety
DORS	Dog Owner Relationship Scale
EPA	Eicosapentaenoic Acid
ESBL	Extended-Spectrum Beta-Lactamase
Fe-BARQ	Feline Behavioural Assessment and Research Questionnaire
GABA	Gamma-Aminobutyric Acid
GAD-7	Generalized Anxiety Disorder-7
IRIS	International Renal Interest Society
LAPS	Lexington Attachment to Pets Scale
LNAA	Large Neutral Amino Acids
MCTs	Medium-Chain Triglycerides
NSAIDs	Non-Steroidal Anti-Inflammatory Drugs
OA	Osteoarthritis
PHQ-9	Patient Health Questionnaire-9
PSS	Perceived Stress Scale
Trp	Tryptophan
Tyr	Tyrosine
